# The Cardiovascular Disease Continuum: From Cardiovascular Risk Factors to Heart Failure

**DOI:** 10.3390/life16020308

**Published:** 2026-02-11

**Authors:** Mariana Floria, Anca Victorita Trifan, Daniela Maria Tanase

**Affiliations:** Department of Internal Medicine, “Grigore T. Popa” University of Medicine and Pharmacy, 700115 Iasi, Romania; floria.mariana@umfiasi.ro (M.F.); daniela.tanase@umfiasi.ro (D.M.T.)

## 1. Introduction

Over the past 30 years, cardiovascular diseases (CVD) have become the dominant contributor to the global disease burden, accounting for approximately 93% of prevalence, 54% of mortality, and 60% of disability-adjusted life-years lost [1]. A CVD continuum represents a successive evolution from cardiovascular risk factors through atherosclerosis and target organ damage (heart, brain, kidneys), and finally, heart failure (HF). Managing risk factors like dyslipidemia, or more specifically hypercholesterolemia and/or hypertriglyceridemia, is crucial to break the cycle to atherosclerosis, endothelium dysfunction, vascular pathology and CVD [1]. Right ventricular (RV) dysfunction and atrial fibrillation (AF) are essential prognosis markers in patients with CVD. Atherosclerosis initiates a cascade of structural- and functional changes that can ultimately progress to HF, with AF emerging as both a consequence and a driver of this evolution. In parallel, RV dysfunction—once considered a secondary phenomenon—has gained recognition as a critical determinant of symptoms, prognosis, and therapeutic response. In CVD, a continuum is essential to know and to explore the pathophysiological links from atherosclerosis to cardiac disease with HF-, AF-, and RV dysfunction having a pivotal role in shaping disease progression and clinical outcomes.

## 2. Cardiovascular Risk Factors as First Step in Cardiovascular Disease Continuum

Cardiovascular risk factors—such as hypertension, hypercholesterolemia, diabetes, and smoking—initiate a gradual progression toward overt CVD. Early risk-factor identification and management can slow or prevent progression along the CVD continuum. Cumulative exposure to risk factors drives structural and functional cardiovascular changes, including atherosclerosis, myocardial remodeling, and arrhythmias. Hypercholesterolemia is one of the most common risk factors encountered in CVD.

Hypercholesterolemia plays a central role in the formation of lipid plaques, particularly through elevated levels of low-density lipoprotein (LDL) [2], thereby increasing the risk of CVD, peripheral arterial disease, and cerebrovascular disease. Early diagnosis and timely intervention to reduce LDL-cholesterol levels are therefore critical for lowering cardiovascular risk. Hypercholesterolemia continues to be a key determinant in the development of atherosclerotic CVD and its associated complications, underscoring the need for sustained and individualized management approaches. Established lipid-lowering therapies, particularly statins, ezetimibe and PCSK9 inhibitors, remain fundamental for reducing LDL-cholesterol levels and mitigating cardiovascular risk when applied in accordance with evidence-based risk stratification and international clinical guidelines [2]. Nonetheless, important treatment gaps remain, especially among patients at very high cardiovascular risk who fail to reach recommended lipid targets. Bridging these gaps requires an integrated strategy that combines lifestyle modification with pharmacogenomic insights and pharmacoeconomic considerations to optimize treatment effectiveness and support long-term adherence [2].

Cardiovascular diseases, particularly atherosclerosis, have increasingly been linked to allergic inflammation [3]. Beyond classical inflammatory pathways, growing evidence suggests that effector cells involved in allergic responses may influence the formation, progression, and instability of coronary atherosclerotic plaques. This interplay underscores the importance of immune-mediated mechanisms in CVD progression. Allergic inflammation—characterized by Th2 cytokine signaling, eosinophil activation, mast-cell degranulation, and alterations in lipid metabolism—can promote endothelial dysfunction, sustain vascular inflammation, and accelerate plaque development [4]. Although allergic conditions such as asthma and atopic dermatitis have been associated with elevated cardiovascular risk, the magnitude and consistency of these relationships differ according to disease type and severity [3]. Notably, much of the existing evidence is observational, and mechanistic understanding in humans remains limited, with many insights derived from preclinical studies. Nonetheless, emerging therapeutic strategies, including cytokine inhibitors, mast-cell stabilizers, and targeted biologics, offer promising avenues for simultaneously modulating allergic inflammation and reducing cardiovascular complications [3].

Aortic stiffness is beyond the cardiac risk-factors, being a marker of target organ-modification in CVD, which is a step forward in the CVD continuum. Among the many predictors of cardiovascular mortality in community populations and in patients with chronic kidney disease, aortic stiffness has been extensively investigated over recent decades and is now recognized as an independent predictor of future cardiovascular events and all-cause mortality [5]. Clinically, aortic stiffness reflects adverse changes in arterial-wall properties that lead to altered vascular diameter and flow, ultimately predisposing individuals to CVD. Aortic pulse wave velocity is considered the gold-standard method for assessing aortic stiffness, with carotid–femoral pulse wave velocity being the most widely used and validated technique. In chronic-kidney-disease patients, aortic stiffness can be modulated by a range of renal- and inflammation-related biomarkers, involving both chronic kidney disease—mineral and bone disorder pathways—and non-chronic kidney disease-related mechanisms. Decorin, a small leucine-rich proteoglycan, is involved in key physiological and pathophysiological processes, including collagen organization, extracellular matrix interactions, growth-factor signaling, and receptor tyrosine kinase modulation [6]. Emerging evidence suggests a link between decorin and atherosclerosis in patients receiving chronic peritoneal dialysis. In this population, advanced age, elevated systolic blood pressure and triglyceride levels, and reduced serum decorin concentrations seem to be independently associated with increased aortic stiffness, with an inverse relationship between log-transformed decorin levels, and with carotid–femoral pulse wave velocity [6]. Low serum decorin levels appear to independently predict the presence of aortic stiffness in patients undergoing chronic peritoneal dialysis. In addition, decorin may represents a novel biomarker for the incorporation into cardiovascular risk stratification models in this high-risk population. Its integration could support the development of targeted therapeutic strategies, facilitate clinician–patient discussions regarding cardiovascular risk, enhance lifestyle interventions in high-risk individuals, and inform decision-making related to pharmacologic therapy or further evaluation for atherosclerotic CVD.

Coronary microvascular dysfunction is increasingly recognized as a multifaceted condition arising from complex molecular and structural interactions, with oxidative stress and inflammation playing central roles, and is characterized by impaired coronary blood flow [7]. This dysfunction is associated with adverse cardiac outcomes, and growing evidence indicates that abnormalities of the coronary microcirculation are present across a wide spectrum of cardiovascular disorders. For decades, myocardial systolic function has been evaluated noninvasively using conventional echocardiographic parameters. More recently, advanced techniques such as global longitudinal strain and myocardial work–derived indices have enabled the detection of subclinical myocardial ischemia and early left ventricular systolic impairment, particularly in patients with preserved ejection fraction. Despite its clinical relevance, noninvasive evaluation of coronary microvascular dysfunction remains challenging. Several imaging modalities—including positron emission tomography, computed tomography, cardiac magnetic resonance, myocardial contrast echocardiography, and transthoracic Doppler echocardiography—can quantify coronary flow reserve and myocardial blood flow, which are key markers of microvascular function [7]. Nevertheless, each technique is constrained by issues related to accessibility, standardization, and diagnostic performance [7]. The close relationship between coronary microvascular dysfunction and myocardial systolic impairment underscores coronary microcirculation as a potential therapeutic target and highlights microvascular indices as promising prognostic markers in diverse cardiovascular conditions [8]. Consequently, incorporating the routine assessment of coronary microcirculation into clinical practice may enhance risk stratification, particularly in patients with conditions such as HF with preserved ejection fraction and angina with non-obstructive coronary arteries [8].

Genetic factors can play a significant role in the development and progression of CVD. In alkaptonuria, a rare inherited disorder of tyrosine metabolism, pigment deposition frequently affects the aortic valve, leading to valvular degeneration and dysfunction [9]. In such complex cases, the Heart Team approach is particularly valuable, as it allows careful balancing of risks and benefits across available therapeutic options tailored to the individual patient. Data on the outcomes of transcatheter aortic valve implantation in patients with alkaptonuria remain limited [9]. Nevertheless, available evidence suggests that procedural outcomes in this population are generally favorable, although individual success is largely determined by the extent of valvular involvement and the patient’s overall clinical status [9].

## 3. Atrial Fibrillation in Cardiovascular Continuum

Atrial fibrillation often represents both a consequence and a driver of CVD progression. It contributes to adverse remodeling, thromboembolic risk, and worsening HF and amplifies morbidity and mortality across the spectrum of CVD. Atrial fibrillation can serve as a prognostic marker, identifying patients at a higher risk for cardiovascular events and complications. Early detection and management of AF are crucial to interrupt the continuum from structural heart disease to HF and stroke.

Chronic coronary syndrome and AF are among the most common CVD worldwide and frequently coexist in clinical practice, where their combination amplifies the risks of morbidity and mortality [10]. These conditions share several underlying pathophysiological mechanisms, including systemic inflammation, endothelial dysfunction, and neurohormonal activation, which promote both arrhythmogenic and atherothrombotic processes [10]. In this context, AF-related clinical scores—such as CHA_2_DS_2_-VA, HAS-BLED, and C2HEST—may represent practical and readily available tools for the early identification of significant coronary artery disease and for overall cardiovascular risk stratification [11]. Derived from routinely collected clinical variables, including age, hypertension, diabetes, HF and prior vascular disease, these scores are inexpensive, simple to apply, and well-suited to everyday clinical use. Their incorporation into diagnostic and decision-making pathways may enhance patient triage and optimize resource allocation, particularly in healthcare settings with limited access to advanced imaging techniques. In both emergency and outpatient environments, where immediate coronary imaging may not be feasible, scores such as HAS-BLED and C2HEST could assist in identifying high-risk individuals who may benefit from early referral for invasive coronary evaluation [11]. Notably, the study by Oancea A.F. and colleagues demonstrated that risk scores originally designed for AF management—especially HAS-BLED and C2HEST—were associated with angiographic coronary artery disease severity, as reflected by their correlations with the Gensini and SYNTAX scores [11].

Chronic obstructive pulmonary disease is associated with heightened systemic inflammation, increased arterial stiffness, platelet activation, endothelial dysfunction, and hypoxia-related oxidative stress, all of which contribute to an elevated risk of cerebrovascular events [12]. Patients with coexisting chronic obstructive pulmonary disease and AF face a markedly higher risk of ischemic stroke, estimated to be approximately 2.85 times greater than in those without this combination [12]. Moreover, individuals with chronic obstructive pulmonary disease have a 1.3-fold increased risk of developing AF compared with those without chronic obstructive pulmonary disease, a risk that may double during periods of frequent disease exacerbation [12]. The occurrence of new-onset AF in patients with chronic obstructive pulmonary disease has also been linked to a 1.75-fold increase in ischemic stroke risk within one year [11]. Notably, recent data from a Chinese population indicate that the coexistence of chronic obstructive pulmonary disease and AF is associated with an almost six-fold increase in ischemic stroke risk [13]. Given that chronic obstructive pulmonary disease is both preventable and treatable, strategies aimed at preventing and optimally managing chronic obstructive pulmonary disease and AF are crucial for reducing the burden of ischemic stroke [13]. Healthcare providers should therefore remain particularly vigilant in identifying and addressing this high-risk population.

Patients with AF, chronic kidney disease, and HF often present with a complex interplay of comorbidities and risk profiles that pose significant challenges in both cardiology and internal medicine practice [14]. These conditions frequently coexist, forming a high-risk triad that substantially increases morbidity and mortality through shared mechanisms such as neurohormonal dysregulation, fluid overload, and chronic inflammation [14]. Commonly used risk-stratification tools, including CHA_2_DS_2_-VASc and HAS-BLED, fail to fully reflect the clinical complexity of patients with multiple coexisting conditions. In particular, traditional scores do not adequately account for multimorbidity, as evidenced by observations showing an inverse association between metabolic comorbidities and stroke-risk scores, as well as a lack of significant correlation between hypertension severity and HF symptom burden [15]. Moreover, neither hypertension nor other prevalent comorbidities appear to consistently align with the degree of HF-related symptoms, suggesting limitations in the current assessment frameworks [15]. These findings highlight the necessity of a personalized, multimodal approach to care in this vulnerable population. Additionally, the absence of meaningful differences in hospital length-of-stay across HF phenotypes emphasizes the need for individualized inpatient management strategies, focusing on early mobilization, optimized volume control, and coordinated treatment of coexisting conditions to improve outcomes [15].

## 4. Right Ventricular Dysfunction in Cardiovascular Continuum

The presence of RV dysfunction is associated with increased morbidity and mortality, being a prognostic marker for hospitalization, adverse events, and response to HF therapies, independent of left ventricular function. Right ventricular dysfunction is a complex clinical syndrome resulting from structural or functional cardiovascular abnormalities that compromise the ventricle’s ability to fill adequately or eject blood efficiently. Right heart–pulmonary circulation unit is an integrated anatomo–functional system distinguished by high-volume blood flow, low intravascular pressures, and minimal pulmonary vascular resistance. Dysfunction of this unit presents a significant clinical challenge, as it may arise from a variety of pathological conditions, each with distinct manifestations. Hemodynamic changes affecting RV pressure and volume can lead to symptomatic presentations, with dyspnea being the most common of HF [16]. The underlying causes of RV dysfunction can be classified based on the specific pathophysiological mechanisms involved. The assessment of RV dysfunction includes a multimodality imaging approach which should incorporate standard and advanced echocardiography, lung ultrasounds, computed tomography, nuclear imaging, and invasive techniques such as right heart catheterization [16].

Aortic stenosis affects approximately 12.5% of elderly patients [17]. In those undergoing surgical aortic valve replacement for severe aortic stenosis, the presence of RV dysfunction—assessed noninvasively via echocardiography—serves as a strong adverse prognostic marker [17]. For elderly patients over 75 or those at elevated surgical risk (EuroSCORE II ≥ 8), transcatheter aortic valve implantation is the preferred intervention [17]. Imaging parameters indicative of RV dysfunction—such as pulmonary artery dilation, RV outflow tract thickening, and RV enlargement—measured via computed tomography have been shown to be robust predictors of 1-year mortality in severe aortic stenosis patients undergoing transcatheter aortic valve implantation [18]. These computer tomography-derived metrics, which can be readily obtained by the operator, provide valuable prognostic information, helping to identify high-risk patients and guide procedural planning with enhanced caution.

## 5. Heart Failure in Cardiovascular Disease Continuum

Heart failure often represents the final stage of progressive CVD, integrating the cumulative impact of risk factors and structural heart damage. The development of HF marks a critical transition from subclinical cardiovascular dysfunction to symptomatic, high-risk disease. Primary- and secondary prevention of HF together with early recognition and management of HF are essential to interrupt the continuum and reduce morbidity and mortality. Heart failure poses a growing public-health challenge, with a prevalence that continues to rise globally [19]. This chronic condition affects millions, causing substantial morbidity and mortality while significantly impairing quality of life. Despite therapeutic advances, HF remains a leading cause of hospitalization and death, with a five-year mortality rate surpassing that of many common cancers [19].

The emergence of de novo HF with acute decompensation often signals rapid disease progression and heightened risk of complications, highlighting the need for close clinical monitoring and timely intervention. The amino-terminal fragment of type B natriuretic peptide (NT-proBNP) has become a key biomarker in managing both chronic and de novo HF [19]. It is widely used for risk stratification, differential diagnosis, and monitoring treatment response. In patients experiencing a first hospitalization for de novo HF with reduced left ventricular ejection fraction, NT-proBNP levels at discharge are inversely correlated with the likelihood of myocardial recovery [20]. Specifically, a greater reduction in NT-proBNP from admission to discharge is associated with a higher probability of myocardial improvement during follow-up [20].

Carbohydrate antigen 125 (CA-125), a mucin traditionally used in the diagnosis and monitoring of ovarian cancer, has recently attracted interest in the context of HF. Elevated CA-125 levels have been linked to HF decompensation and congestion, indicating a potential role in disease monitoring and prognosis [21]. However, its clinical utility in HF remains debated. While some patients with decompensated HF show high CA-125 levels at admission, others exhibit normal values despite severe clinical deterioration [21]. This variability suggests that CA-125 expression in HF is influenced by multiple factors, including clinical status, laboratory findings, echocardiographic parameters, and pharmacologic therapy [21]. Recent evidence indicates that the absence of CA-125 elevation in patients admitted for acute HF is associated with sinus rhythm, sleep apnea–hypopnea syndrome, low NT-proBNP levels, and more than 50% inspiratory collapse of the inferior vena cava [22].

Extracorporeal membrane oxygenation has become an important life-saving intervention for critically ill patients. In cases of drug-induced severe cardiac failure, extracorporeal life support—particularly veno–arterial extracorporeal membrane oxygenation —offers a viable means to maintain circulatory and pulmonary function while supporting spontaneous drug clearance and facilitating myocardial recovery. Extracorporeal membrane oxygenation can therefore serve as a critical hemodynamic support strategy in drug-induced circulatory collapse [23]. Most reported cases have utilized peripheral extracorporeal membrane oxygenation, with survival outcomes showing variable, but generally promising, results [24]. Nevertheless, despite its potential to rescue patients from otherwise fatal toxic cardiomyopathy, the precise role and optimal use of extracorporeal membrane oxygenation in this context remain incompletely defined [24].

In conclusion, the CVD continuum highlights the progressive interplay from atherosclerosis to CVD complicated by AF and/or RV dysfunction, and ultimately HF, emphasizing the interconnected nature of cardiac pathophysiology. Early identification and management of risk factors along this continuum are critical to prevent downstream complications and improve long-term outcomes. Atrial fibrillation and RV dysfunction serve both as markers and mediators of disease progression, underscoring their prognostic significance in cardiovascular care. Integrating multimodal diagnostic approaches, including imaging and biomarker assessment, can enhance risk stratification and guide timely therapeutic interventions. Personalized strategies targeting each stage of the continuum may reduce morbidity and mortality while improving quality of life for patients with progressive CVD. Understanding this continuum reinforces the need for comprehensive, multidisciplinary management to interrupt the cascade from subclinical vascular disease to overt HF. The heterogeneity and multifactorial nature of CVD necessitate individualized therapeutic strategies. In recent years, conventional treatment approaches have been complemented by advances in “omics” technologies—including genomics, metabolomics, epigenomics, proteomics, transcriptomics, and microbiomics—which together enhance our understanding of disease pathophysiology and support the development of personalized medicine.

## Data Availability

Not applicable.
